# Manpower, financial, material resources, and participation level in national fitness: a fuzzy-set QCA approach

**DOI:** 10.3389/fpubh.2024.1375930

**Published:** 2024-10-03

**Authors:** Ping Xu, Liang Zhu, Liu Yang, Jin Yan

**Affiliations:** ^1^College of Physical Education and Health Sciences, Zhejiang Normal University, Jinhua, China; ^2^School of Education, College of Human and Social Futures, University of Newcastle, Newcastle, NSW, Australia; ^3^School of Physical Education and Sports Science, Soochow University, Suzhou, China

**Keywords:** manpower, financial, material resources, participation level in national fitness, fsQCA analysis

## Abstract

**Background:**

In the era of economic globalization, major public health events—such as the Ebola epidemic, the avian influenza epidemic, the “SARS” epidemic, and the COVID-19 pandemic—occur more frequently, severely endangering human life safety and health and making global public health governance a major survival issue of universal concern. Therefore, governments have included the improvement of national health in their sustainable development goals, and its important position in global and national health policies has become increasingly prominent. However, the most common non-medical intervention taken by the government is to encourage people to actively participate in physical fitness activities to prevent disease and improve health. Therefore, how to improve the level of participation in national fitness is not only a hot topic in the academic community, but also a work content that governments around the world attach great importance to.

**Objectives:**

To reveal the complex interaction of the factors affecting the participation level in national fitness and obtain the linkage and adaptation mode of multiple conditions.

**Study design:**

Starting from the three elements of manpower, financial, material resources, combined with the characteristics of the organizational behavior of local governments in China, this study puts forward an integrated analysis framework to understand the difference of the participation level in national fitness in various provinces and regions.

**Methods:**

Fuzzy-set Qualitative Comparative Analysis (fsQCA) is used to carry out configuration analysis on the participation level in national fitness in 31 provinces and regions in China.

**Results:**

First, no single necessary condition can explain the results, whether for high-or low-level national fitness participation. Second, the construction of social sports instructors, public financial support for mass sports, and the supply of sports venues are the core conditions for improving the participation level in national fitness. Third, high-level participation of national fitness is carried out in five ways in China’s provinces.

**Conclusion:**

These findings enrich the literature on improving the participation level in national fitness, and provide useful practical enlightenment for the local governments to increase the participation level in national fitness.

## 1 Introduction

In the era of economic globalization, major public health events—such as the Ebola epidemic, the avian influenza epidemic, the “SARS” epidemic, and the COVID-19 pandemic—occur more frequently, severely endangering human life safety and health and making global public health governance a major survival issue of universal concern. Therefore, governments have included the improvement of national health in their sustainable development goals, and its important position in global and national health policies has become increasingly prominent (1, 2). However, the most common non-medical intervention taken by the government is to encourage people to actively participate in physical fitness activities to prevent disease and improve health (3, 4).

The Chinese government has always attached great importance to national health and regarded it as a human-centered problem (5). As early as 1995, the Chinese government issued the “Outline of the National Fitness Program” to encourage broad public participation in sports and fitness activities. In 2014, “National Fitness” was incorporated into the national strategy, which fully highlighted the status of national fitness in China’s development. In addition, the “Healthy China 2030” Planning Outline issued in 2016 further proposed to “give full play to the positive role of scientific fitness for all in health promotion, chronic disease prevention and rehabilitation” (6). China’s Survey Bulletin on the Status of National Fitness Activities in 2020 shows that the proportion of residents aged 7 and above who regularly participate in physical exercise in 2020 is 37.2%, and the proportion of people who regularly participate in physical exercise increase by 3.3 percentage points compared with the 2014 survey. Participation in physical fitness activities has become one of the important ways and means to improve the quality of life of urban residents in China (7, 8). Therefore, how to improve the participation level in national fitness is not only a hot topic in academic circles, but also a work content to which Chinese local governments attach considerable importance.

However, in a multi-task environment, how the local governments can reasonably and effectively allocate resources to improve the participation level in national fitness, narrow the gap in all aspects of national fitness, and achieve national health remains a major problem that puzzles the high-quality development of national fitness in the country. In view of the unbalanced distribution of resource reserves and factor endowments that can be used to improve the participation level in national fitness among local governments in China, managers must accurately identify the multiple conditions and synergies that affect such participation, and on this basis, combine the resources of local governments to effectively select adaptive optimization strategies. Given this problem, many scholars have carried out in-depth thinking and beneficial exploration. For example, system dynamics has been used to point out that increasing the funding for mass sports and increasing the proportion of stadiums that are open to the public are the driving mechanisms for China to develop regular participation in physical training among the population (9, 10). The government and society (including various social organizations, groups, and units) also have a strong guiding role in mass sports participation through longitudinal intervention experiments (11). Semi-structured interviews were carried out with 14 parents who received KidSport funding in eastern Canada in the previous 2 years, and found that the funding program was conducive to improving the level of children and adolescents’ participation in sports activities (12). Other studies showed that supporting sports organizations to give priority to solving the problem of left-behind children, especially women, is conducive to increasing the participation level in sports activities (13). The above studies focus more on the influence mechanism of single factors, and less on the analysis of core conditions and their combination. In addition, no in-depth explanation is carried out in the “operable action plan to improve the participation level in national fitness,” making it impossible to systematically reveal the complex operation mechanism among multiple factors. Strategies, and other research fields (14, 15). In particular, the configuration perspective has become a popular theoretical construction view in recent years, and has been applied in the following areas: strategic groups, typology, and classification (16); resource configuration (17); relationship between strategy formulation, environment, and strategy configuration and performance (18); and entrepreneurial orientation, configuration of social capital within and outside the industry, and the performance of new enterprises (19). Configuration perspective mainly analyzes the interdependence of cause conditions and the multiple concurrent causal relationships formed by different combinations (20) with equivalent configurations. In other words, the configuration perspective reveals the configuration effect among the important conditions that affect results through an overall analysis and case comparison, and finds the multiple combination paths that produce the desired outcomes and achieve the common purpose (21). In addition, adopted from the configuration perspective, the Qualitative Comparative Analysis (QCA) method combines the advantages of qualitative and quantitative analyses. From this point of view, the multi-dimensional and holistic characteristics of configuration analysis facilitate the analysis of public affairs decision-making issues.

Based on current background, this study empirically explores the driving path for the improvement of the participation level in national fitness in provincial areas from the configuration perspective under the practical scenario of improving the participation level in national fitness in various provinces and regions in China. Specifically, this study seeks to answer the following questions: What is the condition configuration that can improve the participation level in national fitness at the provincial level in China, and which conditions play a more critical role in such participation? To achieve this goal, we combine the three elements of manpower, financial, and material resources in the management of public affairs, as well as the characteristics of the organizational behavior of local governments in China to construct an integrated configurational framework. The participation level in national fitness at the provincial level in China is determined by using QCA through fuzzy sets on a sample of 31 provinces and regions in China. Beyond the particularity of a single case, this study reveals the mechanism of condition configuration that leads to the varying participation levels of national fitness, thus deepening the rational understanding of the path and driving mechanism for its improvement in the provinces. In addition, this study discusses the synergy effect of multiple conditions in the “manpower–financial–material resources” framework, explains the complex interactive nature of multiple conditions behind the improvement of the participation level in national fitness in China’s provinces, and provides new empirical materials and analytical ideas while bridging the gaps in existing studies.

## 2 Configurational framework

**Figure 1:**
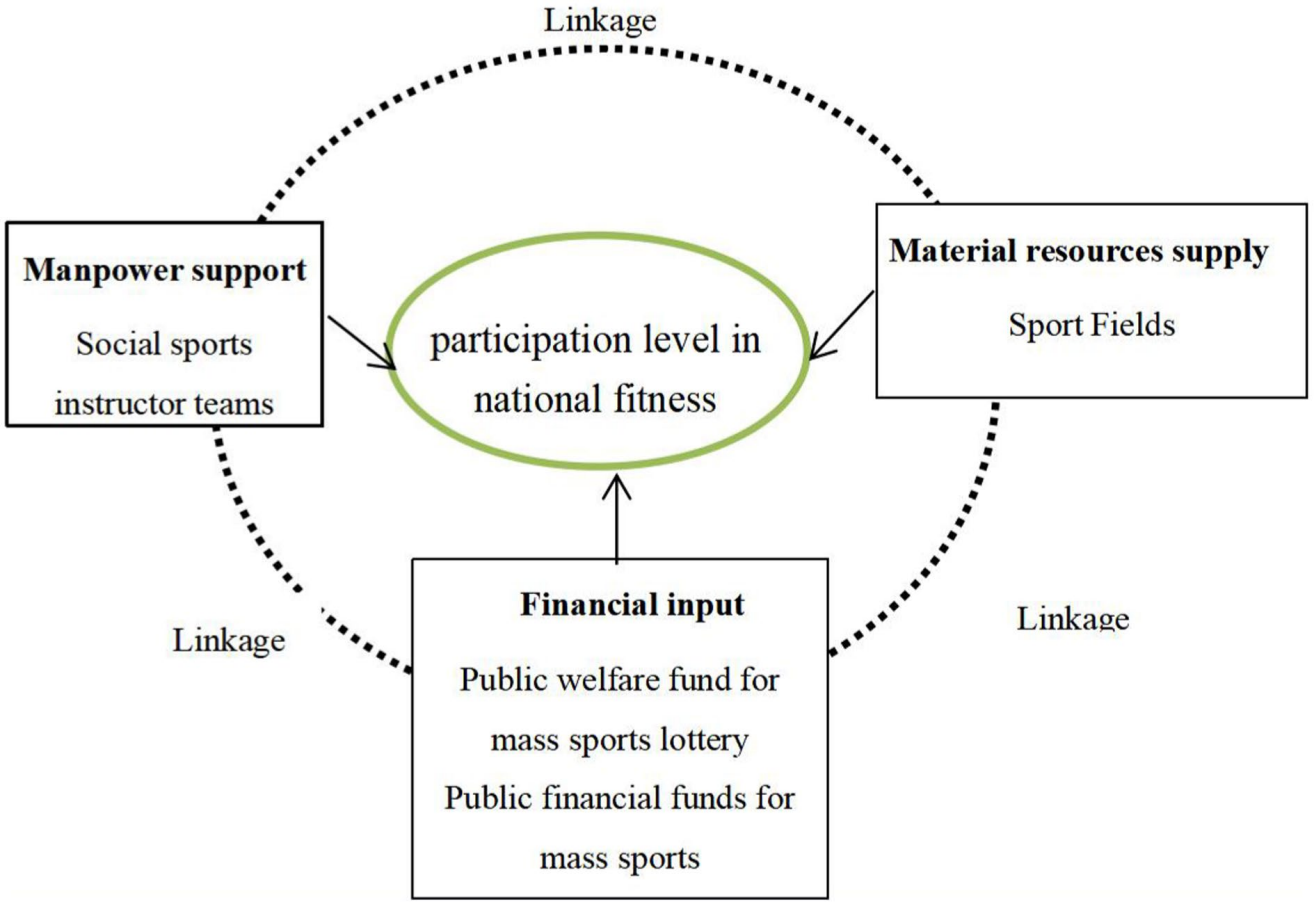
The configurational framework of participation level in national fitness.

From the beginning of the promulgation of the “National Fitness Plan” in 1995 to the implementation of the “Opinions on Building a Higher Level of Public Service System for National Fitness” in 2022, the strategic and fundamental position of national fitness in the construction of a sports power and even a socialist modernization power with Chinese characteristics has increased in prominence. However, the advancement of anything cannot be separated from the three elements of manpower, financial, and material resources (22). Fitness for all is a large-scale social livelihood project led by the Chinese sports administrative department and covers all citizens. Its main work is to implement relevant national policies and regulations on fitness for all. By building a public service system for fitness to meet the needs of the masses, the project can scientifically guide the public to participate in sports and form an active and healthy lifestyle, and ultimately achieve the goal of improving health (23). In this regard, based on the perspective of manpower, financial, and material resources in public affairs management, combined with the institutional situation of China’s provincial government and the practical scenario of improving the participation level in national fitness, this study constructs a “manpower–financial–material resources” configurational framework that is consistent with this study (Figure 1).

The manpower support condition includes two secondary conditions: social sports instructor teams and sports social organizations. Social sports instructors, as personnel engaged in teaching sports skills, fitness guidance and organization management, are important human resources for the development of China’s national fitness. These instructors are basically volunteers. Sports social organizations mobilize all social forces through the integration of social capital, and rely on their own and others’ professional talent team to undertake government sports projects to provide services for the public. Through joint operation, the two meet the requirements of national fitness based on the whole population and life cycle (24). In promoting the development of national fitness in China, the number of social sports instructors and the strength of sports social organizations are increasing, providing a strong human resource support for the extensive development of national fitness activities and the overall improvement of the scientific level of national fitness in China.

The financial input condition includes two secondary forms, namely, public welfare fund for mass sports lottery and public financial fund for mass sports. In China, the cause of national fitness is a popular livelihood project. With sufficient financial resources, the government can invest in the training of sports talents, construction of sports venues and facilities, and the cultivation of sports social organizations. China’s mass sports is an important measure to implement the strategy of national fitness for all. Improving the efficiency of financial investment in mass sports is conducive to alleviating the imbalance between the people’s growing demand for diversified sports and the supply of public sports services (25). The public welfare fund of sports lottery is one form of the non-tax revenues of the Chinese government, which is dedicated to sports, social welfare, and other social welfare undertakings. With the continuous increase of the sales of sports lottery, the amount of public welfare fund extracted also continually increases, and the its proportion invested in mass sports grows at an average annual rate of 16.76% (26). In summary, a large amount of financial investment can not only provide financial guarantee for the popularization of national fitness, but also promote the rational allocation of various sports resources, which is conducive to improving the participation level in national fitness.

The material resources supply condition includes a secondary form of sport fields. Compared with the people’s growing demand for diversified and multi-level fitness, China’s fitness facilities are still insufficient in total quantity, low quality, structurally imbalanced, and lacking in participation of social forces. The problem of “where to go for fitness” has not been fundamentally solved (27). To break through this bottleneck, the governments’ national top-level design and local solid promotion in recent years have highly improved China’s sports venues in both quality and quantity, which provides a material basis for the improvement of the participation level in national fitness.

## 3 Materials and methods

### 3.1 Fuzzy-set qualitative comparative analysis

QCA is a configurational approach based on set theory and fuzzy algebra (28), and is particularly suitable for studying complex causality and multiple interactions (15). In recent years, QCA has also attracted the attention of Chinese scholars, especially in the research fields of government governance, management performance, and technology application, and findings are emerging. This study adopts the method of fuzzy set QCA (fs/QCA) to carry out empirical test from the configuration perspective, and explore the diversified driving mechanism of high-level participation in national fitness in China’s provincial areas.

QCA, as a new method, not only analyzes the research object statistically but also holistically (29). By taking the whole case as the configuration of conditions (variables), QCA has to some extent integrated the advantages of case and variable studies (30). First, a cross-case comparison is used, and its samples can be large, medium and small to avoid a single analysis. In this regard, the external promotion validity of the empirical results can be ensured to a certain extent on the basis of finding the mechanism of conditions. The second is the analysis from the configuration perspective, which can determine the combination of different conditions with equivalent results, and thus deepen the understanding of the driving mechanism of the varying results in different case scenarios, and can further explore the potential substitution relationship between conditions. Third, in the case of “asymmetric causality,” the combination of conditions that lead to the emergence and disappearance of results can expand the theoretical interpretation of specific research issues. In this regard, multiple cases are used on the basis of logical conditions to compare different cases within the same model and between different models (31), and thus learn how to match different conditions to achieve better results (21).

### 3.2 Sample and data collection

In China, national fitness is one of the important contents of sports work in every provincial region. After nearly 30 years of exploration, each provincial region has accumulated rich practical experience, laying a theoretical foundation for researchers in the field of national fitness in China. For this study, we selected all provincial regions (31 in total) in Chinese Mainland as the sample. The data are derived from the 2021 Statistical Yearbook of Sports Undertakings compiled by the Economic Department of the General Administration of Sport of the People’s Republic of China (the data published in this yearbook is 2020), and the “National Fitness Implementation Plan (2021–2025)” issued by each provincial region. Each case has 8–15 pages of information, and provides sufficient qualitative data on the participation in national fitness. The data accuracy is ensured by collecting easily available supplementary materials. The reason for choosing the data from 2020 is that this year marks the closing of China’s “13th Five-Year Plan” and the acceptance of the “National Fitness Implementation Plan (2016–2020).”

### 3.3 Measurement

#### 3.3.1 The outcome is the participation level in national fitness

For China, which has a large population, a small increase in the proportion of people engaging in exercise is already a huge digital progress, but actually reflects the fact that Chinese people really take sports or exercise as a way of life (32). In this regard, we take the proportion of the number of people who regularly participate in physical exercise in Chinese Mainland in 2020 as the measurement index of the level of national fitness participation in the provincial areas.

#### 3.3.2 Social sports instructor teams

With the increasing number of people participating in the national fitness program, many health risk events have also occurred, such as sports injuries and related diseases, leading to threats to people’s health (33). The possible reason is the lack of professional guidance and corresponding safety knowledge when people participate in physical exercise. As a professional fitness guidance team for the public, social sports instructors play a vital role in improving the quality of national fitness development. Therefore, we choose the number of social sports instructors per 1,000 people in provincial areas of Chinese Mainland in 2020 as the measurement index of fitness guidance level.

#### 3.3.3 Sports social organizations

Sports social organizations are not only the basic organizational form for the public to participate in sports activities, but also the indispensable provider of public services for national fitness and the important carrier of national fitness management. The construction of sports social organizations is conducive to facilitating the achievement of national fitness goals, and improving the physical quality and health level of the whole population (34). In this regard, we choose the number of sports social organizations (with legal qualifications) per 10,000 people in each provincial region of Chinese Mainland in 2020 as the measurement index of the strength of sports social organizations.

#### 3.3.4 Public financial funds for mass sports

China’s sports industry is mainly composed of sports competitions, sports training, mass sports and stadiums (35). In existing literature, the proportion of financial expenditure on public sports services to local financial expenditure is used to reflect the intensity of the former (36). On this basis, we take the proportion of public financial investment in mass sports in provincial areas of Chinese Mainland in the total public financial investment in sports in 2020 as the measurement index of public financial support for mass sports.

#### 3.3.5 Public welfare fund for mass sports lottery

According to the Measures for the Administration of Lottery Public Welfare Funds issued by the Ministry of Finance of the People’s Republic of China, the public welfare funds for sports lottery refers to the specified proportion of funds that China withdraws from the sales of sports lottery for the development of sports undertakings. The public welfare funds for sports lottery are turned over to the provincial government, of which the financial department, together with the civil affairs and sports departments, studies and determines the distribution. As stated above, we take the proportion of the amount of sports lottery public welfare funds used in mass sports in provincial areas of Chinese Mainland in the total amount of sports lottery public welfare funds used in 2020 as the measurement indicator of the support of mass sports lottery public welfare funds.

#### 3.3.6 Sport fields

As the material carrier for the public to participate in physical exercise, sports venues are an important support for the development of national fitness. The strong demand for sports venues and its severe shortage of supply *per capita* seriously restrict the development of national fitness activities in China (9). Therefore, to make up for the shortcomings of the national fitness facilities and facilitate the obvious improvement of the fitness environment are necessary, which is conducive to the formation of a good atmosphere for the masses to participate in physical fitness nationwide. Thus, we select the *per capita* sports field area of each provincial area in the Chinese Mainland in 2020 as the measurement index of the field resource support.

### 3.4 Calibration

Calibration involves transforming variables into set membership, ranging from full non-membership that equals 0 to full membership that equals 1; 0.5 is the crossover point and indicates maximum ambiguity (28, 37, 38). Calibration determines the position of a certain value and its specific meaning, that is, to which extent the value belongs to the set of high or low scores. Three direct techniques are used to determine the qualitative anchors for scale measurement (28, 39). First, we use a large amount of knowledge of pre-verified proportional anchors as a threshold (40). However, when certain knowledge is available, the second calibration method is based on the sample maximum, average (or median), and minimum (15, 41). The third technique uses the percentile of the sample in the absence of substantive knowledge (42, 43). The calibration of membership scores of fuzzy sets must be based on a comprehensive analysis of the theoretical knowledge and empirical evidence (9). We adopt the third technique to calibrate the variables. This calibration method can reflect meaningful standards and capture changes directly related to our research issues and target case sets. For the selection of three anchor points, namely, “full membership,” “intersection,” and “full non-membership,” we take 95, 50, and 5% of the sample data, respectively, by referring to existing research practices and case data characteristics (20). To avoid theoretical difficulties in the point of maximum ambiguity (0.5), we add a small constant of 0.001 in accordance with established practices (15, 28) (The calibration result is shown in Table 1).

**Table 1:** Calibration.

Condition			Calibration		
			Fully in	Crossover point	Fully out
Outcome	Participation level in national fitness	Proportion of people who often take part in physical exercise	0.468	0.362	0.323
Antecedent	Manpower	Social sports instructor teams	3.220	2.270	1.435
		Sports social organizations	2.015	0.334	0.209
	Financial	Public welfare fund formass sports lottery	0.118	0.070	0.027
		Public financial funds for mass sports	0.309	0.192	0.071
	Material resources	Sport Fields	2.865	2.150	1.615

## 4 Results

### 4.1 Necessity conditions analysis

Before carrying out a specific path analysis, checking whether any single condition is necessary for participation level in national fitness is necessary. If the consistency coefficient is higher than 0.9, the antecedent can generally be regarded as a necessary condition for the result (44). Table 2 presents the results of this analysis. In the context of high and low levels of proportion of people who often engage in physical exercise, the consistency coefficients of all of the conditions are below 0.9, indicating that no single condition is necessary for participation level in national fitness (28, 37). Therefore, taking a configurational perspective is essential.

**Table 2:** Necessity analysis of single conditions.

Condition	High levels of proportion in people who often take part in physical exercise		Not-high levels of proportion in people who often take part in physical exercise	
	Consistency	Coverage	Consistency	Coverage
Social sports instructor teams	0.677	0.740	0.566	0.568
~Social sports instructor teams	0.605	0.602	0.741	0.678
Sports social organizations	0.630	0.745	0.654	0.711
~Sports social organizations	0.756	0.704	0.766	0.655
Public welfare fund formass sports lottery	0.699	0.740	0.591	0.575
~Public welfare fund formass sports lottery	0.599	0.614	0.733	0.691
Public financial funds for mass sports	0.638	0.685	0.666	0.658
~Public financial funds for mass sports	0.6810	0.689	0.681	0.634
Sport Fields	0.686	0.785	0.529	0.557
~Sport Fields	0.613	0.586	0.795	0.670

### 4.2 Sufficient solutions

As the core of the QCA method, configuration analysis aims to investigate how different combinations of antecedents affect the results. We use fsQCA3.0 software to analyze standardized data. Based on previous research and in combination with the specific situations in the present study, we select a consistency threshold of 0.8 (42, 43), frequency threshold of 1 (15, 45), and a PRI (Proportional Reduction in Inconsistency) threshold greater than or equal to 0.65 (46). Using these comprehensive standards, we obtain the truth table that meets the requirements and the configuration paths through the operation data. Table 3 shows the results. We identify five pathways that can lead to high proportions of people who engage in physical exercise. The overall solution consistency is 0.883, which explains the significance level of all configurations as a whole. The results show that the four configurations capture 74.4% of the high proportion of people who often engage in physical exercise.

**Table 3:** Configurations strongly related to participation level of national fitness.

Antecedent condition	High levels of proportion of people who often take part in physical exercise				
	1	2	3	4	5
Social sports instructor teams (SSI)		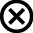	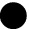	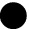	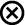
Sports social organizations (SSO)	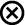	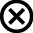	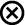	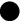	
Public financial support intensity of mass sports (PF)	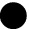	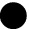	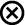		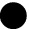
Support intensity of public welfare fund for mass sports (WF)	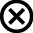	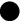		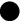	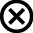
Sport field (SF)			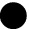	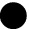	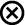
Raw coverage	0.417	0.349	0.346	0.352	0.357
Unique coverage	0.040	0.110	0.072	0.040	0.023
Consistency	0.890	0.921	0.882	0.944	0.890
Overall solution coverage	0.744				
Overall solution consistency	0.883				

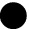
, core casual condition (present); 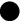, peripheral casual condition (present); 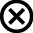, core casual condition (absent); 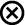, peripheral casual condition (absent). Blank spaces indicate “do not care”.

Configuration 1 is SSO * PF * ~ WF. In this situation where public financial support for mass sports is dominant, if the provincial government can strengthen the construction of sports social organizations, then the region can achieve a high level of regular participation in sports although the support intensity of public welfare funds for mass sports is low. This path has a coverage of 0.417, which means that it can explain approximately 41.7% of the cases in which the participation level in national fitness has improved. In addition, approximately 4.0% of the cases can only be explained by this path, such as Fujian and Liaoning.

Configuration 2 is ~SSI * ~ SSO * PF * WF. At the same time, in provincial areas where the social sports instructors and sports social organizations are relatively weak—that is, the supply of manpower resources is insufficient—if the support of public finance for mass sports and public welfare funds for mass sports lottery can be increased, then the proportion of people who often participate in physical training in this area can also be improved. This path has a coverage of 0.349, which indicates that it can explain approximately 34.9% of the cases in which the participation level in national fitness has improved. In addition, approximately 11.0% of the cases can only be explained by this path, such as Hebei, Hainan, and Hunan.

Configuration 3 is SSI * ~ SSO * ~ PF * SF. For the provincial areas that have both a sound social sports instructor system and a high level of *per capita* sports area, even with relatively scarce sports social organizations and insufficient public financial support for mass sports, the participation level in national fitness in this area can still be improved. This path has a coverage of 0.346, which means that it can explain approximately 34.6% of the cases in which the participation level in national fitness is improved. In addition, approximately 7.2% of the cases can only be explained by this path, such as Beijing, Tianjin, and Shaanxi.

Configuration 4 is SSI * SSO * WF * SF. For provincial-level regions with sufficient manpower resources supply and high-level public welfare fund support for mass sports and *per capita* sports area, the participation level in national fitness in the region can be improved regardless of their public financial support for mass sports. This path has a coverage of 0.352, which means that it can explain approximately 35.2% of the cases in which the participation level in national fitness has been improved. In addition, approximately 4.0% of the cases can only be explained by this path, such as Shanghai, Jiangsu, Zhejiang, and Shandong.

Configuration 5 is ~SSI * PF * ~ WF * ~ SF. Under the condition that public financial support for mass sports plays a central role, the region can also achieve a low level of participation in national fitness even with weak of social sports instructors, weak support of public welfare funds for mass sports, and small area of sports venues *per capita*. This path has a coverage of 0.357, which means that it can explain approximately 35.7% of the cases in which the level of participation in national fitness has improved. In addition, approximately 2.3% of the cases can only be explained by this path, such as Guangxi.

### 4.3 Robustness checks

To verify the robustness of QCA results, we use standard methods such adjusting the calibration threshold, changing the consistency threshold, adding or removing cases, changing the frequency threshold, and adding other conditions (47). Referring to the above methods, we use the ensemble relationship and the fit difference of the configuration proposed by Schneider and Wagemann as the judgment criteria (38). First, we reduce the consistency threshold from 0.8 to 0.75, and the analysis reveals that the five groupings are still supported, with a slight decrease in overall consistency and a slight increase in overall coverage. Second, two cases are randomly selected and removed. Given that the solutions remain similar, the main findings are robust.

## 5 Discussion

At present, China is at a new historical starting point, and is of great relevance and value to continue to improve the participation level in national fitness and thus facilitate its high-quality development. This topic is of general interest to the general public, a cause to which the Chinese central government attaches great importance, and is a research hotspot in Chinese academia. However, in reality, variables can hardly work alone and independently of each other. Managers face a problem of integrated practical applications, that is, they need to solve comprehensive problems. However, existing literature has not explored the core conditions affecting the level of national fitness participation and their complex interaction mechanisms in depth, which this study has attempted to do. The configuration analysis reveals that, first, no single condition is necessary to explain the results of both high and low participation levels in national fitness. Second, the construction of social sports instructors, public financial support for mass sports, and sports venue supply are the core conditions for increasing the participation level in national fitness in China, and the cultivation of sports social organizations and public welfare fund support for mass sports are the auxiliary conditions for increasing the level of national fitness participation in China. Third, five paths can be used to improve the low participation level in national fitness in provincial areas of China, that is, five different combinations of conditions can achieve the goal of improving the participation level in national fitness.

China is a vast country, and different regions not only have different geographical environments, but also different economic, cultural, and social development levels. Given this reality, 31 provincial regions in China have different resource reserves and factor endowments and face different challenges in promoting the level of national fitness participation, and thus a “one-size-fits-all” solution is neither feasible nor desirable. This study explores multiple concurrent causes for the improvement of national fitness participation from a configuration perspective, and finds multiple equivalent paths by using QCA. That is, the 31 provincial governments in China do not have to rely exclusively on any single strategy when adopting strategies to increase the participation level in national fitness, but can choose multiple combinations of paths to achieve their goals based on their own resource allocations. Based on the uneven distribution of manpower, financial, and material resources among local governments, a single strategy choice May be easy for certain provincial areas but difficult for others. Therefore, by analyzing the overall fitness participation levels of 31 provincial regions in China and comparing them across cases, we reveal the group effects among important conditions that increase the participation in fitness and identify multiple combinatorial paths that produce desirable outcomes, thus providing more relevant options for local governments to adopt interventions.

## 6 Implication

### 6.1 Theoretical contributions

First, based on the three elements of manpower, financial, and material resources, we construct a theoretical model to improve the participation level in national fitness and carry out configuration analysis, thus revealing the effect mechanism of the level of participation in national fitness. Thus far, most studies focus on single factors, such as the financial support of the government, cultivation of sports social organizations, and the construction of sports venues. We expand research in this field by exploring the mechanism of improving the participation level in national fitness from the overall perspective. The improvement of the participation level in national fitness is driven by the interaction between manpower, financial, and material resources rather than by any single condition. What is unique is that this study reveals the synergy and matching of manpower, financial, and material resources by explaining the complex mechanism that affects this relationship.

Second, we apply the QCA method to the research on the improvement of the participation level in national fitness, and broadened the research method selection of the national fitness theme research. The empirical analysis of the internal mechanism of improving the participation level in national fitness in a single linear regression is difficult. By introducing QCA into the study on the improvement of the participation level in national fitness, the equivalent mechanism of the high-level participation in national fitness is revealed. In short, QCA can expand and optimize the research methods of the theme of national fitness, providing a new research perspective for the study of small and medium-sized samples in the field of national fitness.

### 6.2 Practical implications

First, the observed synergistic effects of the triple conditions of manpower, financial, and material resources reveal the complexity of the factors affecting the participation level in national fitness. This finding also shows that provincial governments can focus on the combination of different conditions from the overall perspective according to their existing conditions and endowments; that is, by linkage and adaptation, we select the driving path “according to local conditions,” design the matching promotion plan, and formulate targeted and timely policies to form a differentiated path to improve the participation level in national fitness. For example, provincial governments can break the constraints of other weak conditions by constantly improving the construction of sports venues and training social sports instructors, which are also core elements to increase the participation level in national fitness.

Second, the linkage and adaptation between different conditions enables provinces to seek multiple ways to improve the participation level in national fitness, rather than relying on a single path as the traditional approach, such as “compensation for lack of people, money for lack of money.” The unique advantages of each provincial region are not so important, and the provincial regions without any congenital benefits can increase the participation level in national fitness through the concurrent synergy of different conditions, that is, each provincial region can achieve the goal of improving the level of participation in national fitness via “reaching the same goal through different ways.”

### 6.3 Limitations and future research

In the study of promoting the participation level in national fitness, although this study has made a breakthrough attempt, certain limitations remain. First, our research purpose is to analyze the complex interaction that increases the participation level in national fitness in China’s provincial areas through cross-case comparison. Although the QCA and relative regression analysis can provide the possibility for further in-depth study of the case, compared with the in-depth longitudinal case study, it cannot answer questions as “why,” “how,” and other issues. In this regard, follow-up research can combine in-depth interviews, participatory observation and other methods, to explain the internal effect of the motivations and strategies of local government actors on the improvement of the participation level in national fitness, and explore its dynamic mechanism with various influencing factors. Second, in terms of the time dimension of case data, the analysis in this study does not cover cross-year case data, that is, only 1-year data are discussed, which undoubtedly limits the interpretation of research conclusions in the diachronic dimension. Third, due to the availability of data, this study measures the conditions of sports social organizations by using the number of sports social organizations per 10,000 people (registered as legal persons), which is clearly inaccurate. In the practice of promoting the level of participation in national fitness, sports social organizations without legal qualifications are also a relatively large force, and thus future research can strengthen the collection of such data. On this basis, a more in-depth discussion can be carried out.

## Data Availability

The original contributions presented in the study are included in the article/supplementary material, further inquiries can be directed to the corresponding author/s.

## References

[1] Reddock J. Seeking consensus on universal health coverage indicators in the sustainable development goals. J Health Serv Res Policy. (2017) 22:178–82. doi: 10.1177/1355819617704676, PMID: 28480749

[2] Daniel RH Stevens GA Hosseinpoor AR Boerma T. Monitoring universal health coverage within the sustainable development goals: development and baseline data for an index of essential health services. The lancet. Glob Health. (2018) 6:e152–68. doi: 10.1016/S2214-109X(17)30472-2 29248365

[3] Janssen I LeBlanc AG. Systematic review of the health benefits of physical activity and fitness in school-aged children and youth. Int J Behav Nutr Phys Act. (2010) 7:40. doi: 10.1186/1479-5868-7-40, PMID: 20459784 PMC2885312

[4] O’Donovan G Blazevich AJ Boreham C Cooper AR Crank H Ekelund U. The ABC of physical activity for health: a consensus statement from the British Association of Sport and Exercise Sciences. J Sports Sci. (2010) 28:573–91. doi: 10.1080/02640411003671212, PMID: 20401789

[5] Zheng J-Y Luan L-X Sun M. Does the National Fitness Policy Promote National Health?—an empirical study from China [J]. Int J Environ Res Public Health. (2022) 19:9191. doi: 10.3390/ijerph19159191 35954550 PMC9368402

[6] Tan X Zhang Y Shao H. Healthy China 2030, a breakthrough for improving health[J]. Global health promotion, (2019), 26:96–9. doi: 10.1177/1757975917743533 29297762

[7] Chen T Hui E C M Lang W. People, recreational facility and physical activity: New-type urbanization planning for the healthy communities in China[J]. Habitat International, (2016), 58: 12–22. doi: 10.1016/j.habitatint.2016.09.001

[8] Zhang Z Wang M Xu Z. The influence of Community Sports Parks on residents’ subjective well-being: A case study of Zhuhai City, China[J]. Habitat International, (2021), 117:102439. doi: 10.1016/j.habitatint.2021.102439

[9] Yang S Xu J Yang R. Research on coordination and driving factors of sports industry and regional sustainable development—Empirical research based on panel data of provinces and cities in eastern China[J]. Sustainability, (2020), 12: 813. doi: 10.3390/su12030813

[10] Ma Y Kurscheidt M. The National Games of China as a governance instrument in Chinese elite sport: an institutional and agency analysis[J]. International journal of sport policy and politics, (2019), 11: 679–699. doi: 10.1080/19406940.2019.1633383

[11] Kelly L. Sports-based interventions and the local governance of youth crime and antisocial behavior[J]. Journal of sport and social issues, (2013), 37: 261–283. doi: 10.1177/0193723512467193

[12] Clark M Costas-Bradstreet C Holt NL Spence JC. Parental perceptions of a national program that funds sport participation for low-income children and youth in Canada. Leis Sci. (2022) 44:1082–98. doi: 10.1080/01490400.2019.1700573

[13] Eime RM Harvey JT Charity MJ Payne WR. Population levels of sport participation: implications for sport policy. BMC Public Health. (2016) 16:752. doi: 10.1186/s12889-016-3463-5, PMID: 27506922 PMC4977647

[14] Miles RE Snow CC. Organization strategy, structure and process. New York: McGraw-Hill (1978).

[15] Fiss PC. Building better causal theories: a fuzzy set approach to typologies in organization research. Acad Manag J. (2011) 54:393–420. doi: 10.5465/amj.2011.60263120

[16] Ferguson TD Ketchen DJ Jr. Organizational configurations and performance: the role of statistical power in ex⁃tant research. Strateg Manag J. (1999) 20:385–95. doi: 10.1002/(SICI)1097-0266(199904)20:4<385::AID-SMJ24>3.0.CO;2-X

[17] Black JA Boal KB. Strategic resources: traits, configurations and paths to sustainable competitive advantage. Strateg Manag J. (1994) 15:131–48. doi: 10.1002/smj.4250151009

[18] Dess GG Lumpkin GT Covin JG. Entre⁃ preneurial strategy making and firm performance: tests of Contin⁃ gency and configurational models. Strateg Manag J. (1997) 18:677–95. doi: 10.1002/(SICI)1097-0266(199710)18:9<677::AID-SMJ905>3.0.CO;2-Q

[19] Stam W Elfring T. Entrepreneurial orientation and new venture performance: the moderating role of intra-and extra-industry social capital. Acad Manag J. (2008) 51:97–111. doi: 10.5465/amj.2008.30744031

[20] Rihoux B Ragin CC. Configurational Comparative methods: Qualitative comparative analysis (QCA) and related techniques Sage (2009). Available at: https://books.google.com/books?hl=zhCN&lr=&id=sAcIYzgO3nkC&oi=fnd&pg=PR1&dq=20.Rihoux,+B.+%3BC.+C.+Ragin.Configurational+Compara%E2%81%83tive+Methods:+Qualitative+Comparative+Analysis%EF%BC%88QCA%EF%BC%89and+Related+Techniques.Sage.(2009).&ots=u_VJh300AC&sig=m8fs2XSZ7_aq8VUuYXBJhkYdLg#v=onepage&q&f=false

[21] Du YZ Jia LD. Histological perspective and qualitative comparative analysis (QCA): a new path for management research. Manage World. (2017) 6:155–67. doi: 10.19744/j.cnki.11-1235/f.2017.06.012

[22] Fang BX. Enhancing the security posture of cyberspace from the perspective of "people, money and materials". J Chin Acad Sci. (2022) 37:53–9. doi: 10.16418/j.issn.1000-3045.20211117006

[23] Lu WY Chen PJ. Research on the connotation, path and institutional mechanism of the deep integration of national fitness and national health. Sports Sci. (2018) 38:25-39+55. doi: 10.16469/j.css.201805003

[24] Xiang ZB Li XT Wang L. Research on community-social sports organizations-social sports instructors linkage operation mechanism. J Beijing Sport Univ. (2017) 40:23–8. doi: 10.19582/j.cnki.11-3785/g8.2017.09.004

[25] Liu C Li YL Qiu P Zhang G. Research on the efficiency of financial investment in mass sports in Central China--based on DEA-Malmquist model. J Chengdu Sport Univ. (2019) 45:43–50. doi: 10.15942/j.jcsu.2019.03.007

[26] Li L Yang X Lan Z Cao X. Research on public financial investment in mass sports in China. J. Capital Institute PE. (2015) 27:196–201. doi: 10.14036/j.cnki.cn11-4513.2015.03.002

[27] Xu Q Dan Y. Exploring strategies to improve the quality and efficiency of tennis in jingshan in the context of rural revitalization[C]//Of Papers Presented at 2024 5th Asia Sport Science Conference (ASSC). (2024).

[28] Ragin CC. Redesigning social inquiry: Fuzzy sets and beyond. Chicago: University of Chicago Press (2008).

[29] Lacey R Fiss PC. Comparative organizational analysis across multiple levels: a set-theoretic approach In: Whetten DA Felin T King BG, editors. Studying differences between organizations: Comparative approaches to organizational research, Emerald Group publishing limited (2009). 91–116. doi: 10.1108/S0733-558X(2009)0000026006

[30] Ragin CC. The comparative method: moving beyond qualitative and quantitative strategies University of California Press (2014). Available at: https://books.google.com/books?hl=zh-CN&lr=&id=2akwDwAAQBAJ&oi=fnd&pg=PR7&dq=30.Ragin,C.C.The+Comparative+method:+moving+beyond+qualitative+and+quantitative%C2%A0strategies%3BUniversity+of+California+Press.(2014).&ots=vfTdLi0_v_&sig=JdZn1uMa3bsCE498Fdhv2FeOiPU#v=onepage&q&f=false

[31] Smith JD Li DH Rafferty MR. The implementation research logic model: a method for planning, executing, reporting, and synthesizing implementation projects[J]. Implementation Science, (2020), 15: 1–12. doi: 10.1186/s13012-020-01041-8 32988389 PMC7523057

[32] Dai J Menhas R. Sustainable development goals, sports and physical activity: the localization of health-related sustainable development goals through sports in China: a narrative review[J]. Risk management and healthcare policy, (2020):1419–1430. doi: 10.2147/RMHP.S257844 32943959 PMC7478917

[33] Yang SQ. Research on the risk and governance of national health sports. J Guangzhou Sport Univ. (2017) 37:10–6. doi: 10.13830/j.cnki.cn44-1129/g8.2017.03.003

[34] Ma JH Shu WP Wang J. Study on the improvement of national fitness organization network and effectiveness in western cities--Xi'an City, Shanxi Province as an example. J Sports. (2021) 28:85–90. doi: 10.1623/j.cnki.cn44-14-4/g8.20201209.002

[35] Qiu P Li YL Liu C. Research on financial investment in public sports services in China: scale, structure and efficiency. J. Chengdu Sport Univ. (2019) 34:105–12. doi: 10.13297/j.cnki.issn1005-0000.2019.02.003

[36] Zhu P. A study on the efficiency of financial expenditure on public services of sports in 31 provinces of China based on DEA. J Sports. (2022) 29:66–71.

[37] Schneider CQ Wagemann C. Set-theoretic methods for the social sciences: A guide to qualitative comparative analysis. Cambridge: Cambridge University Press (2012).

[38] Kraus S Ribeiro-Soriano D Schüssler M. Fuzzy-set qualitative comparative analysis (fsQCA) in entrepreneurship and innovation research-the rise of a method. Int Entrep Manag J. (2018) 14:15–33. doi: 10.1007/s11365-017-0461-8

[39] Du YZ Kim PH. One size does not fit all: strategy configurations, complex environments, and new venture performance in emerging economies. J Bus Res. (2021) 124:272–85. doi: 10.1016/j.jbusres.2020.11.059

[40] Misangyi VF Greckhamer T Furnari S Fiss PC Crilly D Aguilera R. Embracing causal complexity: the emergence of a neo-configurational perspective. J Manag. (2017) 43:255–82. doi: 10.1177/0149206316679252

[41] Ordanini A Maglio PP. Market orientation, internal process, and external network: a qualitative comparative analysis of key decisional alternatives in the new service development. Decis Sci. (2009) 40:601–25. doi: 10.1111/j.1540-5915.2009.00238.x

[42] De Crescenzo V Ribeiro-Soriano DE Covin JG. Exploring the viability of equity crowdfunding as a fundraising instrument: a configurational analysis of contingency factors that lead to crowdfunding success and failure. J Bus Res. (2020) 115:348–56. doi: 10.1016/j.jbusres.2019.09.051

[43] Ribeiro-Navarrete S Palacios-Marques D Lassala C Ulrich K. Key factors of information management for crowdfunding investor satisfaction. Int J Inf Manag. (2021) 59:102354. doi: 10.1016/j.ijinfomgt.2021.102354

[44] Thomas G Santi F Fiss PC Aguilera RV. Studying configurations with qualitative comparative analysis: best practices in strategy and organization research. Strateg Organ. (2018) 16:482–95. doi: 10.1177/1476127018786487

[45] Bell RG Filatotchev I Aguilera RV. Corporate governance and investors’ perceptions of foreign IPO value: an institutional perspective. Acad Manag J. (2014) 57:301–20. doi: 10.5465/amj.2011.0146

[46] Greckhamer. T.CEO compensation in relation to worker compensation across countries: the configurational impact of country-level institutions. Strateg Manag J. (2016) 37:793–815. doi: 10.1002/smj.2370

[47] Zhang M Du YZ. Qualitative comparative analysis (QCA) in management and organization research: position, tactics, and directions. Chin J Manag. (2019) 16:1312–23. doi: 10.1016/j.jbusres.2015.10.125

